# The Effects of Chain Conformation and Nanostructure on the Dielectric Properties of Polymers

**DOI:** 10.3390/ma18010198

**Published:** 2025-01-05

**Authors:** Gabriel Mogbojuri, Shaghayegh Abtahi, Nayanathara Hendeniya, Boyce Chang

**Affiliations:** Department of Materials Science and Engineering, Iowa State University, Ames, IA 50011, USA

**Keywords:** dielectric polymers, breakdown strength, dielectric constant/permittivity, dielectric loss, nanocomposites, nanofillers, nanostructure, energy density, chain conformation

## Abstract

The dielectric properties of polymers play a pivotal role in the development of advanced materials for energy storage, electronics, and insulation. This review comprehensively explores the critical relationship between polymer chain conformation, nanostructure, and dielectric properties, focusing on parameters such as dielectric constant, dielectric loss, and dielectric breakdown strength. It highlights how factors like chain rigidity, free volume, molecular alignment, and interfacial effects significantly influence dielectric performance. Special emphasis is placed on the impact of nanofillers, molecular weight, crystallinity, and multilayer structures in optimizing these properties. By synthesizing findings from recent experimental and theoretical studies, this review identifies strategies to enhance energy efficiency, reliability, and mechanical stability of polymer-based dielectrics. We also delve into techniques such as electrostatic force microscopy (EFM) and focused ion beam (FIB) milling for characterizing breakdown mechanisms, offering insights into molecular design for next-generation high-performance polymers. Despite considerable progress, critical challenges such as achieving an optimal balance between dielectric permittivity and breakdown strength, understanding nanoscale interfacial phenomena, and scaling these materials for industrial applications persist. These gaps can be addressed by systematic structure–property relations, advanced processing techniques, and environmental studies.

## 1. Introduction

Polymers have shown tremendous potential across diverse applications, with their role in electronics and energy storage emerging as a rapidly expanding area of focus. This has positioned them as a pivotal subject of research. In particular, polymer dielectric materials have demonstrated versatility in applications such as capacitors [1,2,3,4], transistors [5,6], photovoltaic devices [7,8], and electrical insulation [9,10]. Unlike other dielectric materials, such as ceramics, polymer dielectrics offer exceptional adaptability for molding into various configurations, enabling a broader spectrum of applications. For instance, they are widely used as insulation or semiconducting layers in electrical cables and as dielectric films in capacitors for energy storage in power systems. Their inherent flexibility also makes them valuable in actuator devices, where they can undergo mechanical deformation under an electric field, facilitating efficient energy conversion between electrical and mechanical forms. However, despite these advantages, the low permittivity of polymer dielectrics has hindered their progress in keeping pace with modern technological advancements. This limitation has spurred a growing interest within both industrial and academic circles to develop polymer-based materials with superior dielectric properties [11,12,13,14]. Furthermore, the rising demand in the energy storage sector has reinforced the preference for polymers over ceramics, given their ability to fail safely under high electric fields [15]. These combined factors continue to drive innovations in polymer-based dielectric materials, solidifying their importance in advanced energy and electronic systems.

The selection of polymer dielectric materials for such applications is influenced by a variety of factors, particularly their dielectric properties, including dielectric constant/permittivity (ε′), band gap, dielectric breakdown field, and dielectric loss (ε″). Additionally, physical and chemical attributes, such as morphology, glass transition temperature, and mechanical strength, are often critical considerations. Industrial implementation further depends on cost-effectiveness, making the balance between performance and affordability a key factor [15]. Having a high ε′ enables the polymers to store more energy. This is particularly important in capacitors and other energy storage devices [1,2,16]. The Dielectric breakdown strength (E_BD_) is the maximum electric field a polymer can withstand before catastrophic conduction. Thus, the E_BD_ must be large to ensure safe and energy-efficient dielectrics for energy storage and insulation applications [1,2]. ε″ refers to the energy loss due to heat in an alternating field. This property is essential in high-frequency applications and energy-efficient devices [17].

The energy stored in a capacitor is directly proportional to both the κ and the square of the electric field. Higher κ and E_BD_ enable polymers to store more energy per unit volume. This allows for the scaling of devices into power systems that are smaller, lighter, and more efficient [18]. A low ε″ reduces energy dissipation as heat, thereby enhancing the power cycling efficiency, which is critical for applications like electric vehicles. In addition to these, a high breakdown field extends a dielectric device’s lifespan and improves safety under high-field conditions [19]. In addition, the processibility of polymers in general provides a more cost-effective and scalable manufacturing compared to ceramics [20]. Nonetheless, polymers need to withstand high temperatures without degrading, as overheating can lead to failures, especially in electronics and high-power applications. Durable yet flexible polymers are needed to endure mechanical stresses and to facilitate integration into compact or flexible electronic designs. Furthermore, polymers in energy storage and insulation must resist chemical degradation to ensure long-term stability and performance, especially in harsh environmental conditions [21].

These challenges can be overcome through felicitous selection of molecular architecture. Among these structural features, polymer chain conformation significantly influences the alignment, mobility, and interaction of polar groups within the material [22]. For example, flexible chain conformations such as polyethylene (PE) or polypropylene (PP) enable dipoles to align more easily under an electric field, resulting in higher κ. In contrast, rigid chains or those with extensive conjugation, such as in polyimides, exhibit reduced dipole mobility, which can lower the κ while enhancing E_BD_ by resisting deformation and charge transport pathways under high electric fields [22,23]. Chain conformation also affects the degree of crystallinity in polymers, with crystalline regions typically exhibiting lower ε″ due to restricted chain mobility, which reduces energy dissipation as heat. However, fully crystalline structures may experience reduced **ε**′ because of limited polar segment mobility, whereas semi-crystalline polymers achieve a balance, combining reduced ε″ from crystalline regions with higher **ε**′ from the flexible amorphous regions [16,24,25]. Additionally, in polymer nanocomposites, the conformation of polymer chains around nanoparticles influences interfacial polarization, creating unique interfacial regions that enhance dielectric properties. These tightly conforming chains can increase the **ε**′ without significantly increasing ε″, highlighting the potential of nanocomposites for high-performance applications [26,27].

This review focuses on exploring how variations in chain conformation impact these dielectric properties, providing insights into the mechanisms by which conformation and nanostructure affects material performance. By examining conformational influences, such as those seen in amorphous versus crystalline regions, or flexible versus rigid chain structures, we outline recent strategies for tailoring polymer architecture to enhance dielectric properties. Overall, we aim to provide a unique perspective on the interplay between polymer conformation and dielectric behavior, ultimately guiding the design and development of next-generation polymer dielectric materials with superior performance for energy storage, electronics, and insulation applications.

## 2. Relationship Between Chain Conformation and Dielectric Properties

### 2.1. Background

**Figure 1:**
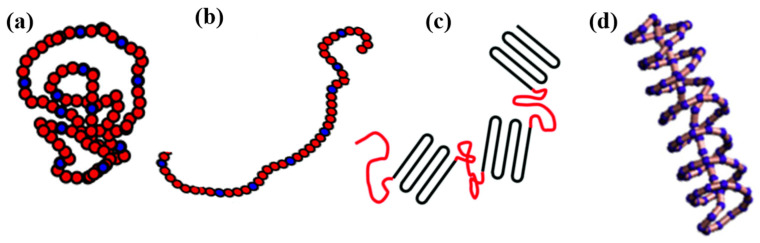
(**a**) Random coil and (**b**) extended chain are adapted from Bryn et al. [37], while (**c**) folded chain conformation is shown based on the work of Faqiang et al. [38], with permission from the Royal Society of Chemistry, and (**d**) helical structure is presented according to Zhiyong et al. [39], with permission from Elsevier.

A polymer’s chain conformation is fundamental in shaping the dielectric properties of materials, directly impacting their effectiveness in applications that demand high electric energy storage and resistance to breakdown [28]. A chain conformation describes the three-dimensional arrangement of atoms within a polymer, which can dynamically shift due to rotations around the bonds in the polymer backbone. This ability to change conformation contrasts with polymer configuration, which is defined by the fixed covalent bonding sequence along the chain and cannot be altered without breaking bonds. This flexibility enables a variety of large-scale polymer conformations, including the following:

Random Coil: Linear chains adopt a random coil, maximizing conformational entropy. This is a flexible and amorphous conformation, where polymer chains take on a disordered arrangement often found in solution and amorphous melts, as shown in Figure 1a. Random coils are often used to describe the extensive molecular overlap and entanglement in polymer melts [29,30].

Extended/Rod-like Chain: In crystalline regions, polymers adopt more ordered, stretched structures (Figure 1b). This type enhances chain packing and often occurs during crystallization or in response to mechanical stretching, leading to an organized, linear form that influences the material’s mechanical strength and stiffness [31,32].

Folded Chain: As shown in Figure 1c, the folded chain structure is commonly observed in semicrystalline polymers; folded chains alternate back and forth, creating layered, lamellar structures. This minimizes the free energy and stabilizes the crystalline regions within the polymer, particularly significant in fibers [30,33].

Helical Structure: Some polymers, especially those with chiral or bulky side groups, adopt helical conformations (Figure 1d). Helical structures provide a specific spatial orientation that can impact optical and dielectric properties, particularly in biopolymers and specialty synthetic polymers [34,35].

#### Factors Influencing Polymer Chain Conformation

Polymer chain conformation is highly sensitive to various external conditions. These influences govern the polymer’s physical properties and suitability for applications.

Temperature: Higher temperatures increase the kinetic energy of polymer chains, allowing greater conformational freedom. This results in expanded chains and a shift toward random coil conformations, particularly in amorphous regions. Conversely, lower temperatures stabilize more ordered structures, such as folded and crystalline conformations [29,34,36].

Solvent Quality: Solvent interactions significantly impact chain conformation. In good solvents, where polymer–solvent interactions are favorable, chains tend to swell and expand. In poor solvents, polymers collapse into denser conformations, favoring entanglement and folded structures. The Flory–Huggins parameter χ helps describe these interactions, where low χ values encourage expanded conformations, and higher values drive chain compaction [30,31,34].

Mechanical Stretching: Applying mechanical force orients polymer chains along the direction of the stress, promoting extended chain conformations. This process is often used in polymer processing (e.g., fiber spinning) to enhance material properties like tensile strength. Stress-induced alignment can increase crystalline content, impacting the material’s rigidity and thermal stability [29,32].

Processing Techniques: Techniques such as extrusion, spinning, or casting impose shear and align chains in specific directions. These methods can induce crystallinity and alignment, leading to extended and folded conformations that improve mechanical and barrier properties in the final material [33,36].

Understanding the conformation of polymer chains is not only critical for grasping the physical structure of polymers but also plays a significant role in determining their dielectric properties. The arrangement of polymer chains affects how effectively a material can store electric energy, withstand electrical breakdown, and manage energy dissipation. For instance, the packing density of chains, alignment of dipoles, and overall molecular order can either enhance or limit the material’s **ε′**, E_BD_, and ε″ [40]. By examining these relationships, we can appreciate how different conformations directly influence a polymer’s suitability for applications in high-energy storage, insulation, and low-loss electronic devices.

### 2.2. Impact on Dielectric Constant (κ/ε_r_/ε′)

**Figure 2:**
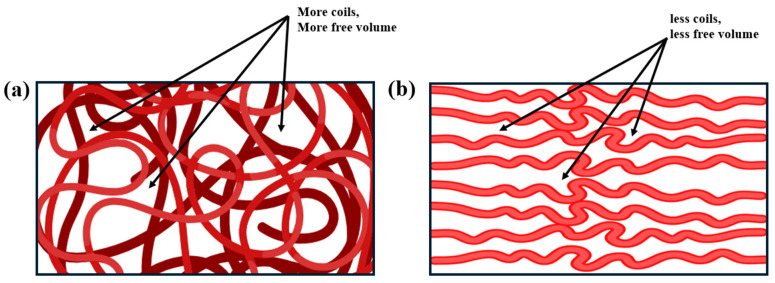

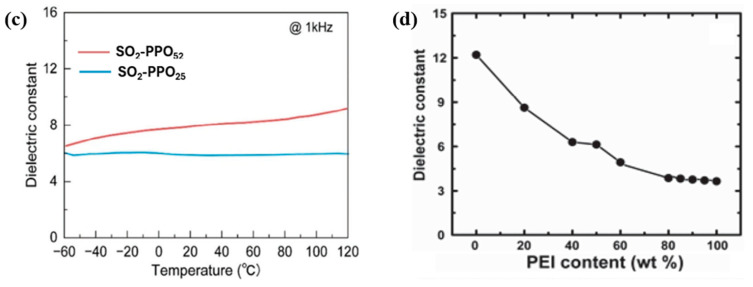
(**a**) Polymer matrix with a less ordered and more coiled conformations showing more free volumes. (**b**) Polymer matrix with more ordered and extended conformations with less free volume. (**c**) Temperature-dependent dielectric constant values of sulfonylated PPOs (SO_2_-PPO_25_ and SO_2_-PPO_52_), adapted from Chen et al., originally reported by Zhang et al. [40,44], Copyright 2023, Wiley. (**d**) Dielectric constant variation as a function of PEI content in PVH/PEI blends, from the study by Ramani et al. [45].

To begin, the concept of free volume in polymers refers to the unoccupied spaces within the polymer matrix, allowing molecular chains to move, rotate, and reorient, directly influencing chain conformation and flexibility [41]. In amorphous regions (Figure 2a) where chains are loosely packed, free volume is higher, enabling segments to adjust their orientations under thermal and mechanical stimuli. This increased flexibility supports adaptable, more conformable chain structures. Conversely, in crystalline domains, chains are more densely packed in extended conformations (Figure 2b), limiting free volume and reinforcing rigidity. Such closely packed arrangements restrict segmental mobility, leading to a more stable, ordered structure [41,42].

Free volume and **ε′** have a complex relationship. Intuitively, polymers with randomly oriented chain structures; hence, higher free volumes tend to lower **ε′**, as these voids introduce regions with dielectric properties closer to air or vacuum [40,43]. However, free volume can also improve the polarizability of the molecule leading to higher **ε′**. Zhang et al. investigated this relationship by exploring strategies to tailor free volume in polymers and probing their dielectric properties. One approach involved designing polymers with a rigid backbone, such as sulfonylated poly(2,6-dimethyl-1,4-phenylene oxide) (SO_2_-PPO), to increase free volume while enabling dipole rotation. In SO_2_-PPO, the rigid backbone provides substantial free volume, allowing for rotational freedom for methylsulfonyl side groups. This configuration minimizes disruptive dipole–dipole interactions, facilitating dipole alignment under an electric field and producing a high **ε′** (**ε′** = 8.2 for SO_2_-PPO_52_), as shown in Figure 2c [44]. This chain rigidity helps stabilize the dielectric response by reducing sensitivity to temperature and frequency fluctuations, making SO_2_-PPO an effective model for enhancing dielectric properties in polar polymers. In contrast, another strategy based on intrinsic microporosity induces conformational distortion, increasing free volume by approximately 10%, reducing dipole density, and thereby lowering the **ε′** [40].

Supporting this, Ramani et al. [45]. developed the relationship between free volume and **ε′** in a fluorine-containing polyimide blend. In a blend of poly(ether imide) (PEI) and poly(vinylidene fluoride-co-hexafluoropropylene) (PVH), they found that increased free volume due to PVH groups facilitated greater dipole alignment, enhancing the ε′, especially at reduced PEI content between 20–80% (Figure 2d). At around 80% PEI, the ε′ stabilizes due to strong dipolar interactions between PEI and PVH, which constrain dipole mobility, producing a controlled dielectric response. Although larger free volumes can improve the ε′ by promoting dipole mobility, excessive free volume dilutes dipole density, reducing ε′. Consequently, controlled adjustments in free volume through chain conformation or structural packing allow precise tuning of dielectric responses, balancing dipole mobility with matrix stability. This approach is invaluable for designing polyimides with high and stable ε′, meeting the demands of electronic applications requiring consistent charge storage and robust dielectric properties.

Nevertheless, Ronova et al. [22] demonstrated that free volume cannot fully describe the trends observed on ε′ of polyimides. Here, the addition of a methyl side groups increased free volume but lead to lower ε′. In contrast, lowering the free volume by altering the backbone chemistry resulted in mixed trends, highlighting a knowledge gap in the structure–property relationship is needed to understand the interplay between rigidity, free volume, and dielectric properties.

### 2.3. Impact on Dielectric Loss (ε″/Tanδ)

At high frequencies, ε″ in polymers is predominantly governed by orientation polarization and dipole relaxation [46,47]. Orientation polarization occurs when permanent dipoles align with an external electric field [48], while dipole relaxation describes the delay in this alignment under rapidly changing fields [49,50]. Rigid polymer chains, which typically adopt extended conformations as established earlier, restrict dipole reorientation and consequently lower ε″ due to constrained dipole mobility [51]. This reduced dipole reorientation is advantageous in applications requiring low ε″, as it indicates a decreased susceptibility of polymer chains to respond to an alternating electric field.

**Figure 3:**
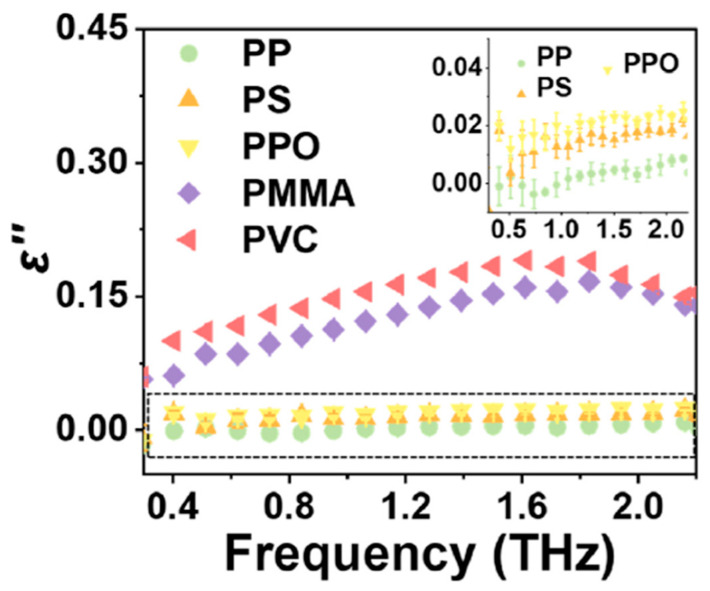
Dielectric loss (ε″) of five distinct samples, plotted as a function of frequency in the THz band, reported with permission from Xiao et al. [52], Copyright 2024, Elsevier.

A focused investigation by Xiao et al. [52] highlights the interplay between chain rigidity and ε″ in five polymers: polypropylene (PP), polystyrene (PS), polyphenylene oxide (PPO), polymethyl methacrylate (PMMA), and polyvinyl chloride (PVC). Using Terahertz Time-Domain Spectroscopy (THz-TDS), they examined the ε″ characteristics of these polymers at terahertz frequencies. Their findings revealed that PVC exhibited the highest ε″ due to the presence of chlorine atoms, which contribute to enhanced orientation polarization and relaxation effects, resulting in a marked increase in ε″ (Figure 3). PMMA also displayed elevated ε″ due to ester groups that intensify polarization. In contrast, the rigid benzene ring backbone of PPO limited chain flexibility, thereby reducing dipole activity and minimizing ε″. PP, characterized by weak polarization, showed the lowest ε″ due to minimal dipole interactions, while PS exhibited a moderately higher ε″ than PP because its phenyl group produces a larger dipole moment, which increases polarization and ε″. This study underscores the influence of conformational elements, such as chain rigidity and specific atomic groups, in defining the ε″ profiles of polymers at terahertz frequencies.

### 2.4. Impact on Dielectric Breakdown Strength (E_BD_)

The dielectric strength is considered one of the governing parameters in determining the electrostatic energy storage density (U_e_) in dielectric materials. This is due to the quadratic dependence of U_e_ on E_BD_, as described by this Equation:

$$U_{e}=\frac{1}{2}\varepsilon_{0}\varepsilon_{r}E_{BD}^{2}$$

where ε_0_ is the vacuum permittivity, ε_r_ represents the dielectric permittivity or dielectric constant, and E_BD_ is the maximum electric field a dielectric can endure without degradation [1,16,53,54,55]. Enhancing E_BD_ is a key strategy for improving power density in capacitors and other energy storage applications.

The conformation of polymer chains significantly impacts their dielectric E_BD_. Ordered chain arrangements, such as extended or crystalline structures, yield tightly packed configurations with reduced free volume, which minimizes charge mobility and inhibits the formation of conductive pathways. This enhances E_BD_ by suppressing dielectric failure mechanisms. In contrast, amorphous or loosely packed structures often feature higher free volume, facilitating charge transport and increasing susceptibility to breakdown under high electric fields [56,57,58].

**Figure 4:**
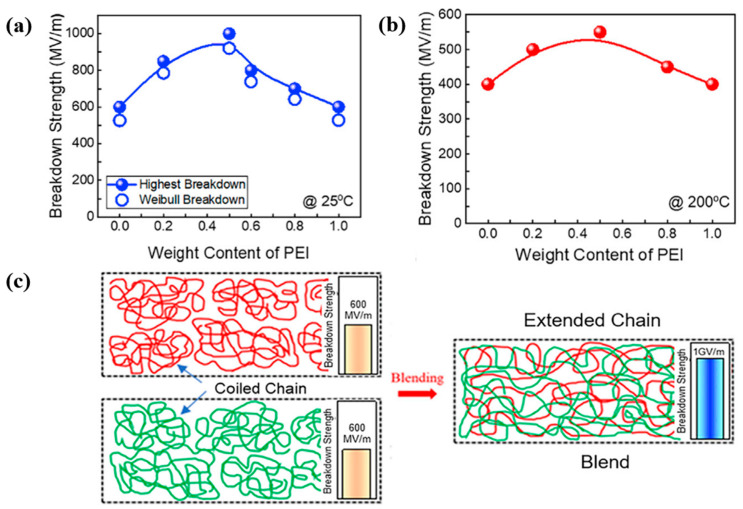

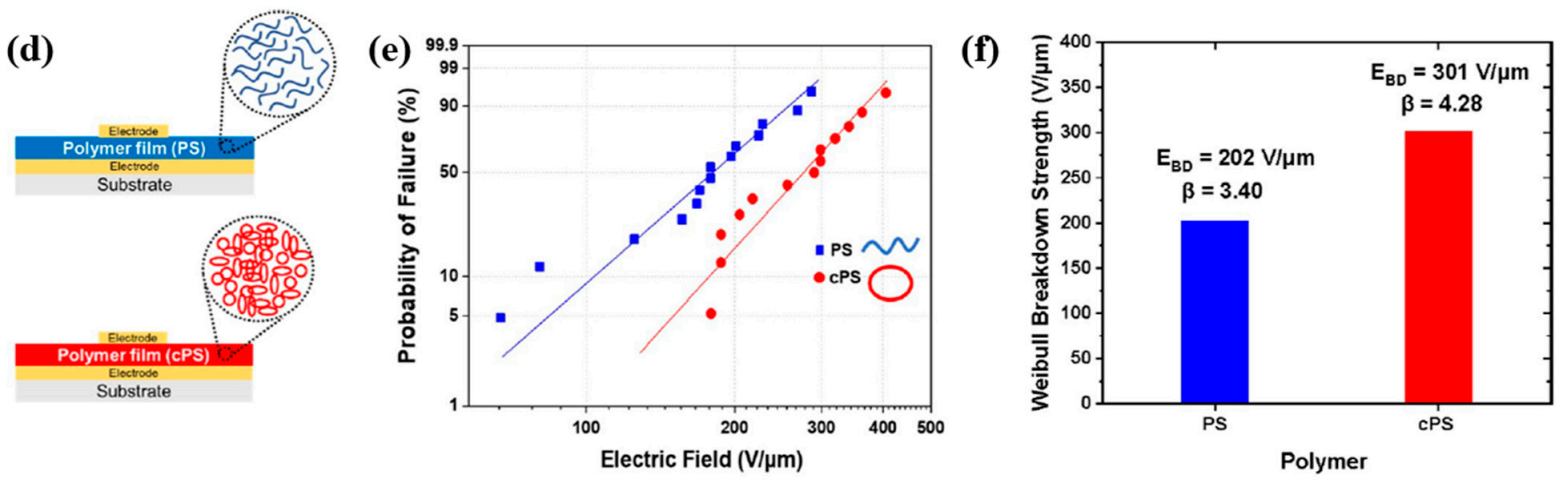
(**a**) Dielectric breakdown strength E_BD_ as a function of the PI/PEI blend ratio at room temperature, including data error margins. Both the highest measured E_BD_ and Weibull E_BD_ values are presented for clarity. (**b**) Breakdown strength at 200 °C, showing the highest E_BD_ achieved. (**c**) A schematic illustration of the PI/PEI blend strategy, highlighting the enhanced electrostatic interactions and the engineering of extended chain packing (data in a, b, and c adapted with Permission from Zhang et al. [28], Copyright 2021, Elsevier). (**d**) Structural schematics of linear polystyrene (PS) and cyclic polystyrene (cPS) films used as dielectric capacitors. (**e**) Weibull failure plots comparing the dielectric reliability of PS and cPS films. (**f**) Weibull breakdown strength (E_BD_), showing ~50% higher EBD for cPS, highlighting its superior dielectric performance. As reported by Singh et al. [55].

A novel approach to improving E_BD_ involves manipulating chain packing density and conformational arrangement of chains via polymer blending. Zhang et al. [28] demonstrated this by blending polyimide (PI) with poly(ether imide) (PEI), achieving strong interchain electrostatic interactions. This method reduced void density and free volume, leading to an extended chain conformation and a 10% reduction in interchain spacing. The optimized 50:50 PI/PEI blend exhibited remarkable E_BD_ values, reaching 1000 MV/m at room temperature (Figure 4a), a 65% improvement over pristine polymers and 550 MV/m at 200 °C (Figure 4b). Molecular dynamics simulations corroborated these findings, revealing reduced mixing energy and microvoids in extended chain conformations (Figure 4c), which enhanced packing density and thermal stability, suggesting a scalable pathway for designing high-performance dielectric polymers for capacitors, electronic devices, and thermal interface materials.

Building on this, Singh et al. [55] demonstrated the advantage of polymer topology by comparing the dielectric properties of cyclic polystyrene (cPS) and its linear counterpart (Figure 4d). Their study revealed that cPS films significantly outperform their linear counterparts in dielectric properties, achieving a 50% enhancement in dielectric E_BD_ (238–300 V/μm for cPS vs. 133–200 V/μm for linear polystyrene) as shown in Figure 4e,f and an 80% increase in capacitive energy density. This improvement is attributed to the elimination of chain ends in cyclic polymers, which reduces defect sites and free volume, thereby enhancing packing density and stability against dielectric breakdown. Here, refractive index measurements were used to substantiate the denser molecular packing. These findings highlight the transformative potential of chain conformation and topology manipulation as strategies to optimize the dielectric properties of polymeric materials, paving the way for advancements in energy storage applications.

The breakdown mechanisms in polymer dielectrics have been the focus of extensive theoretical and experimental investigations, yet a comprehensive understanding remains elusive. Classical theories, such as the intrinsic breakdown model [59], electron avalanche breakdown model [60], thermal breakdown model [61], and electromechanical breakdown model [62], have been proposed to explain these phenomena, and there have also been studies that considers the coexistence and interplay of these model [63]. These mechanisms are complex and intricately linked with the breakdown behavior being influenced by several factors, including material composition, electrical and thermal conductivities, glass transition temperature, operational and environmental temperatures, and the film thickness.

### 2.5. Improved Dielectric Properties at High Temperature

**Figure 5:**
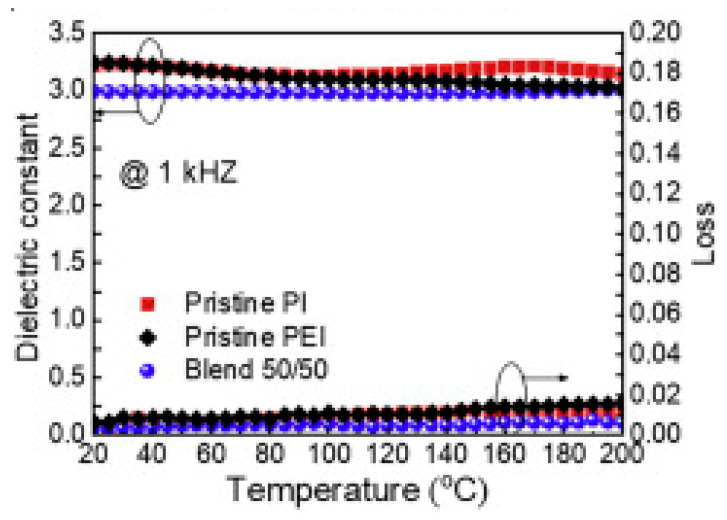
The dielectric constant and dielectric loss of PI, PEI, and the 50–50% blend were measured at 1 kHz across varying temperatures. The data points are presented with solid lines included for visual clarity (the arrow in the figure highlights the specific dataset corresponding to the dielectric property being measured), as reported by Zhang et al. [28], Copyright 2021, Elsevier.

Recent advancements in polymer science have unveiled remarkable dielectric properties at high temperatures, driven by innovative strategies to enhance material performance. Building on the discussion in Section 2.4, Zhang et al. [28] developed an effective method to improve the high-temperature dielectric performance of polymers through precise engineering of chain-packing behavior in polymer blends. By blending high-temperature polymers such as polyimide (PI) and poly(ether imide) (PEI), this approach achieved denser chain packing, significantly reducing voids and free volumes within the material. This optimization led to substantial enhancements in dielectric breakdown strength (EBD), with a 50:50 PI/PEI blend reaching EBD values of up to 1000 MV/m at room temperature and 550 MV/m at 200 °C (Figure 5), representing a 65% improvement at room temperature and a 35% enhancement at elevated temperatures compared to the neat polymers.

Complementing Zhang’s findings, Xudong Wu and Daniel Q. Tan [64] investigated a different strategy to enhance high-temperature dielectric performance by annealing syndiotactic polystyrene (sPS) films. Through annealing near their melting point, the researchers induced the formation of thinner and more numerous lamellae structures, resulting in a refined crystalline architecture. This structural improvement significantly increased the breakdown strength of the material by approximately 19.6% at 120 °C compared to untreated samples. Additionally, annealed films exhibited nearly a 50% reduction in dielectric loss at elevated temperatures, a result attributed to the enhanced crystalline structure’s ability to hinder charge carrier movement, thereby suppressing dielectric loss and expanding the operational temperature range of dielectric polymers. These complementary studies emphasize the transformative potential of molecular and structural engineering techniques—such as polymer blending and annealing treatments—for optimizing dielectric materials in high-temperature applications. Together, they provide valuable insights and practical pathways for developing next-generation materials with superior energy storage capabilities, thermal stability, and electrical performance, advancing the field of high-temperature polymer dielectrics.

## 3. Effect of Nanofillers on Conformation and Dielectric Properties of Polymer

Dielectric polymers exhibit electrical properties due to polarization occurring within their molecular chains, which involves a limited displacement of electron cloud density in the dipoles, leading to the formation of a net dipole moment under an electric field. These polymers have attracted substantial interest and have been utilized across various applications, such as film capacitors, electronic communications, and energy storage materials [16,65,66,67], thanks to their high flexibility, low density, and capability for repeated processing [18]. Dipolar polarization in polymers is influenced by the presence of polar groups and the conformation of the polymer chain. In non-polar polymers, structural symmetry causes dipole moments to cancel out, leading to generally low ε′. Conversely, polar polymers contain permanent dipoles, resulting in permanent dipole moments and, therefore, higher ε′. Enhancing dipolar polarization can be achieved by stimulating dipole mobility, such as by adding polar pendant groups to polymer chains or through copolymerization, prior to reaching dipole saturation [68]. Besides altering the structure of polymer backbones and substituents [69,70,71], ε′ can be tuned by adjusting microstructures with an external field [72,73,74] and forming a composite by loading inorganic fillers [75].

**Figure 6:**
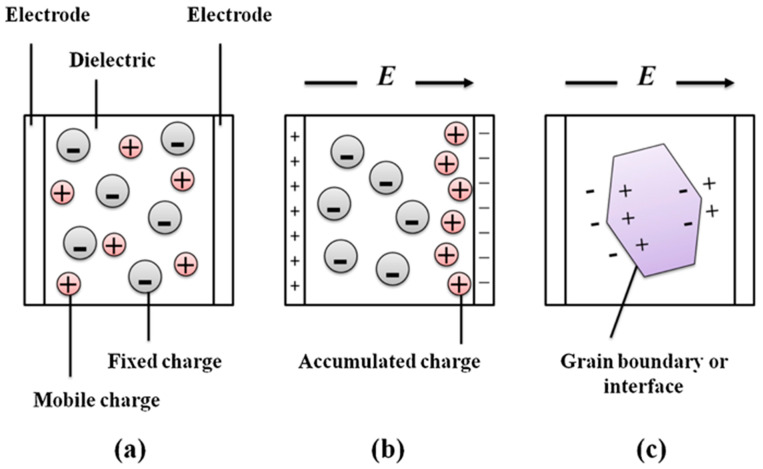
(**a**) A crystal with equal number of mobile positive ions and fixed negative ions. In the absence of a field, there is no net separation between all the positive charges and all the negative charges. (**b**) In the presence of an applied field, the mobile positive ions migrate toward the negative electrode and accumulate there, resulting in an overall separation between the negative charges and positive charges in the dielectric. The dielectric therefore exhibits interfacial polarization. (**c**) Grain boundaries and interfaces between different materials frequently give rise to interfacial polarization. Adapted with permission from Fan et al. [68], Copyright 2019, Elsevier.

The addition of nanoscale fillers to polymer dielectrics to create nanodielectrics results in materials with significantly improved dielectric E_BD_, ε′, and ε″ properties [76]. Nanocomposites can be categorized into two types based on the electrical conductivity of the nanofillers used: dielectric–dielectric nanocomposites, which incorporate semi-conductive nanofillers with high ε′ like ZrO_2_ [77], TiO_2_ [78], Pb(Zr, Ti)O_3_ [79], BaTiO_3_ (BT) [80,81,82], CaCu_3_Ti_4_O_12_ [83], and BN [19,84]; and dielectric-conductive nanocomposites, which contain conductive metal nanofillers such as Ag [85] or Cu [86], conductive polymers like polyaniline [87] or polypyrrole [88], and carbon-based materials, including carbon black [89,90], carbon nanotubes [91,92], and graphite nanoplatelets [93,94]. Using fillers with high ε′ to reinforce a polymer is based on interfacial polarization. Interfacial polarization, or Maxwell–Wagner–Sillars (MWS) polarization [95], arises when positive charges gather at the interface, while remaining negative charges in the bulk create dipole moments within the polarization vector. This effect occurs when there is a charge buildup at an interface between two regions, especially in heterogeneous dielectric materials, as illustrated in Figure 6. In real polymer-based materials, factors such as non-homogeneity, impurities, crystal-amorphous interfaces, and incomplete contact between the film and electrode can contribute to interfacial polarization.

Fan et al. provide a comprehensive review of polymer-based composites for achieving high-energy-density film capacitors [68]. Instead, this Section focuses on the effect of inorganic fillers on the polymer matrix and its resulting dielectric properties.

### 3.1. Effect of Nanofiller Volume and Structure on Dielectric Properties

Achieving simultaneous improvement in ε′ and E_BD_ in nanocomposites remains a significant challenge. High-ε′ nanofillers often fail to substantially enhance nanocomposite performance unless their content is sufficient to achieve mutual connectivity. According to effective medium theory, a volume fraction of at least 25% is required to meaningfully increase dielectric ε′ [96,97]. Incorporating fillers with ε′ over 100 times that of the polymer matrix at high volume fractions (>20%) has been explored to enhance ε′, with the composite ε′ generally representing a geometric average of its components [98,99,100,101,102]. However, even at lower volume fractions (5–10%), the E_BD_ of nanocomposites can decline sharply due to the generation of inhomogeneous electric fields [103]. These fields create localized electrical defect centers, compromising the material’s E_BD_. While effective for enhancing ε′, these fillers render the material unstable for high-energy applications, underscoring the persistent challenge of balancing enhanced dielectric properties with material stability and reliability.

**Figure 7:**
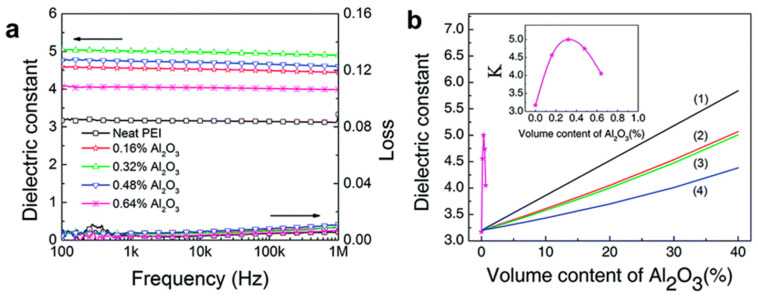

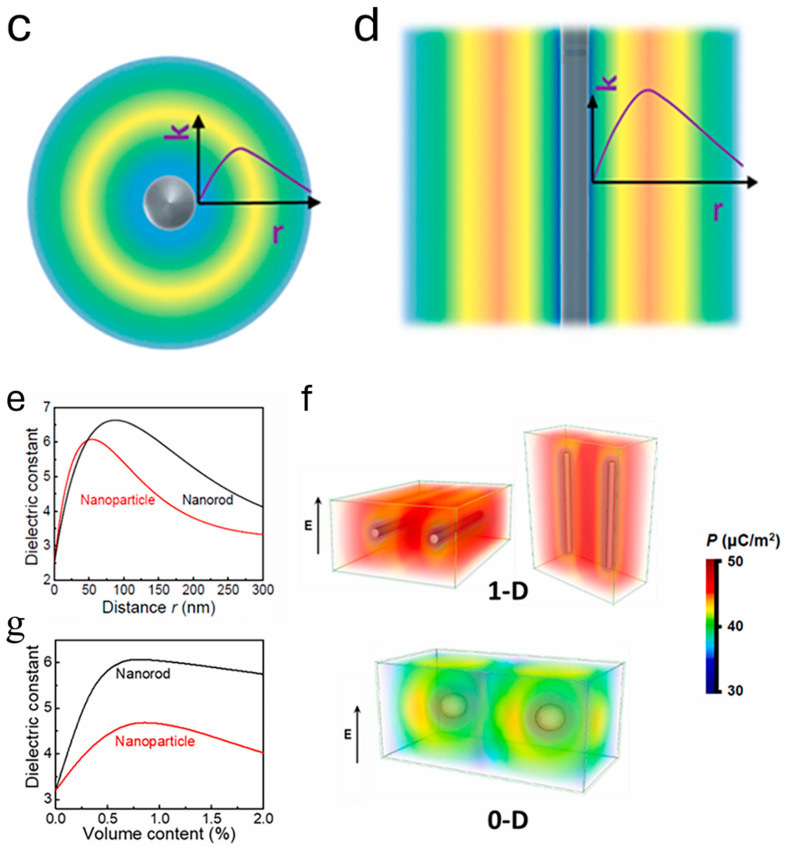
(**a**) Room temperature dielectric properties of PEI/alumina (20 nm particle size) nanocomposites at different alumina nanoparticle loadings (in vol%) vs. frequency. (**b**) Dielectric constant ε′ (at 1 kHz) of nanocomposite films of PEI/alumina (20 nm particle size) vs. nanofiller volume content and comparison with several widely used dielectric models of diphasic dielectric composites (lines with no data points): curves: (1) parallel model, (2) Maxwell model, (3) Lichtenecker model, and (4) series model, see the ESI† for these models. Inset shows an expanded view of the ε′ of the composite films vs. alumina loading. Experimental data points are shown, and lines are drawn to guide the eye. As reported by Thakur et al. [104] with permission from the Royal Society of Chemistry (**c**) Schematic of the ε′ distribution in the interfacial region of a polymer with a spherical shell topology formed by 0-D (nanoparticle) fillers as a function of the distance from the filler surface. (**d**) Schematic of the ε′ distribution in the interfacial region of a polymer with a cylindrical shell topology created by 1-D nanorod fillers as a function of the distance from the filler surface. (**e**) Modeling results of the ε′ distribution in the interfacial region of the polymer as a function of distance (rrr) from the surface of nanofillers for both nanorods and nanoparticles. (**f**) Simulation results of the polarization distribution in the polymer matrix: upper panel shows 3D views for 1-D composites with 35 nm nanorods in series and parallel models, and the bottom panel displays a 3D view for 0-D composites with 50 nm nanoparticles. (**g**) Modeling results comparing the ε′ of PEI nanocomposites with 35 nm nanorods versus filler loading to that of PEI nanocomposites with 50 nm nanoparticles. Adapted from Chen et al. [105] with permission from Elsevier.

To address this, alternative approaches have been explored. Thakur et al. suggest the use of nanofillers with ε′ closer to the polymer matrix, which mitigates intense local electric fields and enhances stability [104]. For instance, adding alumina (Al_2_O_3_) nanoparticles, which possess a relatively low ε′, to a polyetherimide (PEI) matrix significantly improves the composite’s ε′, even at minimal filler volumes as low as 0.32%. This enhancement arises from interfacial effects: the nanofillers modify the dipolar response in the polymer matrix, allowing polymer dipoles to respond more freely to applied electric fields. Smaller nanoparticles (e.g., 20 nm) amplify this effect due to their high surface area, which increases interfacial regions. These interfacial interactions, rather than the inherent dielectric properties of the fillers, drive the observed dielectric enhancement, as shown in Figure 7a,b.

Chen et al. further demonstrates the impact of nanofiller structure and dimensionality on dielectric performance [105]. By comparing zero-dimensional (0-D) nanoparticles with one-dimensional (1-D) nanorods embedded in a PEI matrix, they reveal that even ultra-low filler volumes (<1%) can significantly improve dielectric properties due to extensive interfacial regions. Notably, 1-D nanorods outperform 0-D nanoparticles by achieving a tenfold increase in the dipolar response. This is attributed to the cylindrical shell-like interfacial structures formed by 1-D nanorods, which extend the high-ε′ regions more effectively than the spherical shells of 0-D nanoparticles. These cylindrical structures align better with the applied electric field, reducing the influence of low-dielectric areas and enabling a broader and more substantial dielectric enhancement. Figure 7c,d illustrates the differing shell topologies, while phase-field simulations in Figure 7e–g confirm these findings by showing how nanorods create extended high-ε′ zones.

In summary, the volume and structure of nanofillers are critical to optimizing dielectric properties in nanocomposites. Using low-dielectric fillers like alumina, smaller nanoparticle sizes, and high-aspect-ratio structures such as nanorods leverages interfacial effects to enhance ε′ while minimizing E_BD_ reductions. However, nanofillers and their interfacial regions may distort polymer chain conformation, introducing stress points or localized defects. These disruptions weaken the dielectric strength by amplifying electric field inhomogeneities, underscoring the need for tailored filler designs compatible with the polymer matrix for reliable high-performance materials.

### 3.2. Effect of Nanofiller on Crystallinity and Dielectric Properties

Crystalline properties also greatly impact the energy-storage performance of semi-crystalline polymers, as their aggregation structures—such as crystalline polymorph, average crystal grain size, and overall crystallinity—affect the ε′ and E_BD_ of polymer films [18,106,107]. Polyvinylidene fluoride (PVDF) serves as a prime example of how crystalline structures influence dielectric properties. PVDF exhibits a crystallinity range of 35–70% and features five distinct crystalline polymorphs: α, β, γ, δ, and ε.

Tang et al. [108] investigates the effects of nanofillers, and recrystallization on microstructure, phase transformation, and dielectric properties in PVDF nanocomposites. They found out that as the crystallinity intensifies, the mobility of polymer chains is restricted, reducing the extent of the α-to-β phase transformation. This limited transformation at high crystallinity suggests that achieving an optimal phase composition requires careful control over the crystallization process to balance chain alignment and molecular orientation. Additionally, the addition of carbon nanofibers (CNFs) into the PVDF generates a heterogeneous structure, which also improves the ε′ through MWS polarization. In this process, charges become trapped at the interfaces between the polymer and the conductive nanofillers, leading to increased dielectric response, especially at lower frequencies. Furthermore, the presence of CNFs encourages β-phase crystallization within the PVDF matrix, increasing the ε′ due to the enhanced dipole density associated with the β-phase structure. However, this enhancement effect diminishes with excessive CNF loading. Higher concentrations of CNFs can result in filler aggregation, which disrupts polymer chain mobility and creates localized stress that inhibits the formation of the polar β-phase, ultimately reducing dielectric performance.

**Figure 8:**
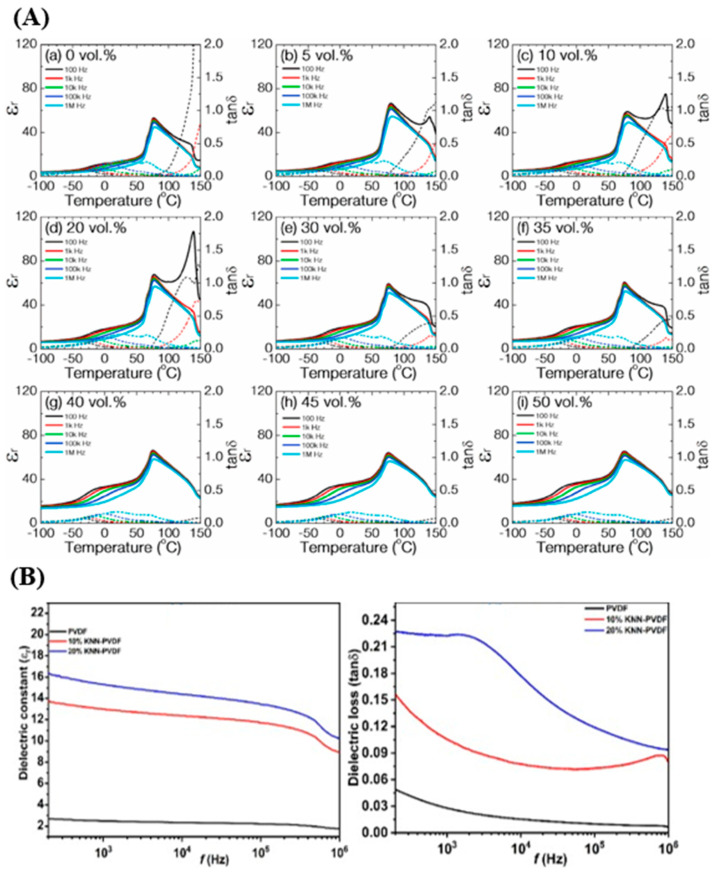
(**A**) Temperature dependence of dielectric permittivity (ε_r_) and dielectric loss (tanδ) for P(VDF-TrFE)-BST nanocomposite films with different BST contents in the cooling measurements as reported by Lu et al. [109]; (**B**) dielectric constant and dielectric loss of PVDF and composite from Mane et al. [110] Copyright 2021, Elsevier.

In another study, Lu et al. [109] investigates how the addition of ceramic nanofillers, specifically barium strontium titanate (BST) nanoparticles, affects the crystallization behavior and dielectric properties of a poly(vinylidene fluoride-trifluoroethylene) (P(VDF-TrFE)) semicrystalline matrix. The researchers found that the presence of BST nanofillers creates two types of crystals in the polymer matrix. Crystals formed on the surface of BST nanoparticles favor the highly ordered β-phase, which contributes to improved dielectric performance. On the other hand, crystals formed within the polymer itself include a mixture of well-ordered β-phase and a defect-rich β′ phase with lower dielectric properties. As the concentration of BST increases, the proportion of defected β′ phase decreases until only the high-dielectric β-phase is present at filler contents above 35 vol%. Dielectric measurements in Figure 8A illustrate that BST nanofillers generally increase ε′, with a particularly significant jump in ε′ occurring between 35% and 40% BST concentration. This jump corresponds to a phase composition shift in the polymer matrix, where the defect-rich β′ phase is largely replaced by the well-ordered β-phase, contributing to the higher dielectric properties observed at these and higher filler levels. This effect confirms that the improved dielectric properties are a result of the crystallization behavior influenced by BST nanoparticles, which guide the formation of favorable polymer phases and optimize interfacial polarization. In another related study, Mane et al. [110] reported the successful hydrothermal synthesis of lead-free potassium sodium niobate (KNN) nano-bricks at low temperatures and their integration into a poly(vinylidene fluoride) (PVDF) matrix to fabricate advanced polymer–ceramic composites. The incorporation of KNN significantly improved the thermal, dielectric, and ferroelectric properties of PVDF. Specifically, at 10% and 20% KNN loading, the remanent polarization of PVDF increased markedly from 0.04 μC/cm^2^ (pristine PVDF) to 6.10 μC/cm^2^ and 7.3 μC/cm^2^, respectively. Additionally, the dielectric constant of the composites exhibited a remarkable enhancement, increasing by 5 to 6 times, albeit with a slight increase in dielectric loss (Figure 8B). These enhancements are attributed to the strong interfacial interactions between the PVDF matrix and KNN nanostructures, which facilitate the promotion of the electroactive β-phase and significantly enhance polarization behavior.

### 3.3. Nanofiller Dispersion and Its Effect on Dielectric Properties

The dispersion of nanofillers in polymer nanocomposites plays a critical role in determining their dielectric properties, as both nanofiller size and surface modification influence their interaction with the polymer matrix. Smaller nanofillers, such as CeO_2_ (<25 nm) and Er_2_O_3_ (<100 nm, crystalline size 8.4 nm), increase surface area, enhance interfacial polarization (IFP), and improve ε′ [111,112]. They also promote better ion mobility and conductivity by creating efficient pathways for ion migration. In contrast, larger nanofillers, like BaTiO_3_ (<100 nm, crystalline size 12.56 nm), can induce matrix inhomogeneities, reducing their effectiveness despite their inherently high ε′.

Surface modification techniques further enhance nanofiller dispersion and compatibility. While chemical coupling agents are widely used, they face challenges like incompatibility with polymer chains, side reactions, and limited stability due to physical adsorption [113]. Polymer grafting offers a superior alternative, producing surfaces with energy profiles more compatible with the polymer matrix. For example, polymethylmethacrylate (PMMA)-grafted BaTiO_3_ nanoparticles in PMMA nanocomposites achieve twice the energy density and extraction efficiency of BaTiO_3_ modified with traditional coupling agents [113].

Dispersion can also be improved through external strategies such as optimized film processing conditions [114], pH adjustments to increase electrostatic repulsion [115], or magnetic field application [116]. Internal strategies like chemical modification or polymer grafting [117,118] further enhance nanofiller integration, ensuring uniform dispersion, reduced ε′ contrast, and improved dielectric performance while maintaining material stability.

### 3.4. Strategies to Mitigate Nanofiller Aggregation in Polymer Matrices

**Figure 9:**
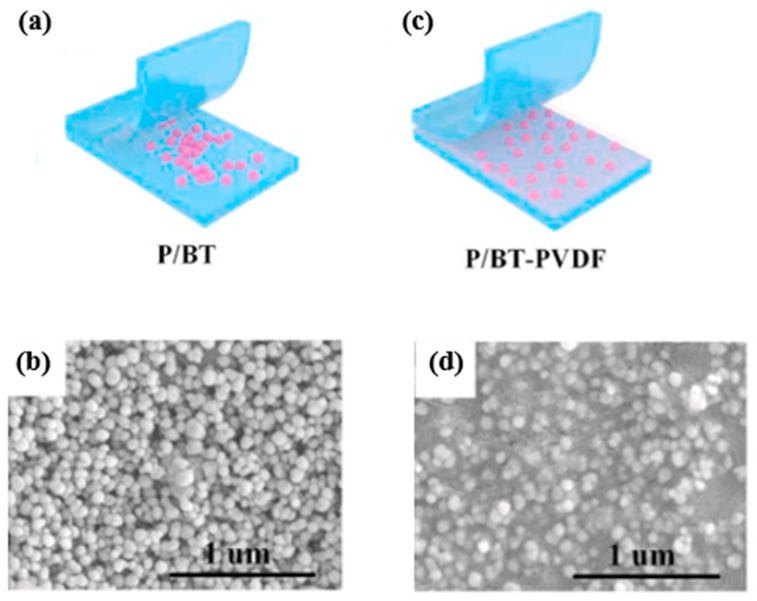
(**a**,**b**) Structure schematic of PMMA/BT and image showing filler aggregation, respectively. (**c**,**d**) The structure schematic of PMMA/BT-PVDF and surface image showing more dispersed fillers, as reported by Zhou et al [119]. Copyright 2021, Elsevier.

One of the primary challenges in incorporating nanofillers into polymer matrices is their tendency to aggregate, which generates localized electric fields and leads to a deterioration in dielectric properties. To address this issue, various strategies have been explored. For instance, Zhou et al. [119] proposed an innovative approach using a sandwich structural design to mitigate filler aggregation. This method incorporates poly(vinylidene fluoride) (PVDF) as a binder for high-dielectric BaTiO_3_ (BT) nanoparticles within a poly(methyl methacrylate) (PMMA) matrix (Figure 9). PVDF serves dual functions: it acts as a physical binder to stabilize the BT nanoparticles and also contributes to the dielectric properties by forming a gradient dielectric constant distribution. This gradient reduces local electric field concentrations, enhances interfacial adhesion, and suppresses aggregation, leading to improved structural stability and uniform filler dispersion. The resulting structure demonstrates significantly enhanced breakdown strength, energy density, and overall electrical performance compared to conventional methods where filler agglomeration is prevalent.

In a related study, Vu et al. [120] tackled the issue of nanofiller aggregation by employing a surface modification strategy using polyethyleneimine (PEI). Here, nanodiamonds (NDs) are pre-treated with a PEI coating, which acts as both a binding agent and a compatibility enhancer between the nanofillers and the polymer matrix. The PEI coating effectively reduces the surface energy of the nanofillers, preventing spontaneous aggregation and promoting better dispersion within the matrix. This strategy not only improves interfacial adhesion but also enhances the material’s functional performance. For example, membranes treated with this method exhibit reduced non-selective voids and improved gas separation selectivity, such as for CO_2_/N_2_ separation. This study highlights PEI’s dual role as a dispersion stabilizer and functional agent, offering a promising pathway for improved nanocomposite integration and application. A summary comparing dielectric properties of polymer and nanocomposites is provided in Table 1.

## 4. Effect of Nanostructure and Thickness Dependence

The dielectric properties of polymer films are significantly influenced by their thickness and nanostructure. Factors such as film thickness, multilayer designs, and periodic structures collectively play a role in determining key parameters such as ε′, ε″, and E_BD_. This Section first examines the dependence of dielectric properties on film thickness, then explores the effects of multilayer structures, and a discussion of advanced characterization techniques used to analyze these relationships.

### 4.1. Effect of Thickness on Dielectric Properties

**Figure 10:**
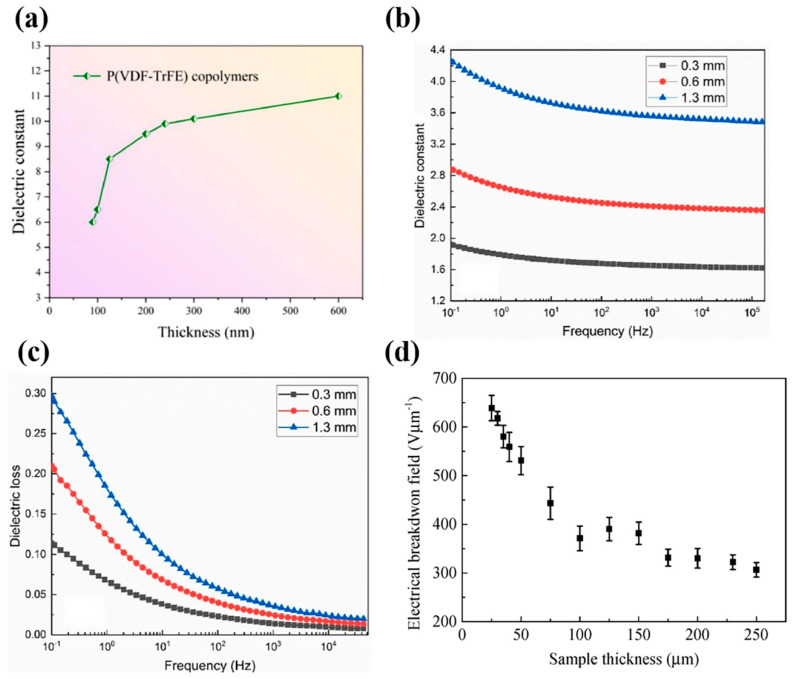
(**a**) Dielectric constant of P(VDF-TrFE) copolymer as a function of film thickness, measured at 1 kHz, adapted with permission from Xudong et al. [121], Copyright 2022, Elsevier, originally reported by Zhang et al. [122]. (**b**) Effect of material thickness on dielectric constant at varying frequency. (**c**) Influence of varying sample thicknesses dielectric loss, as presented by Wang and Yang [123], Copyright 2023, Elsevier. (**d**) Electric breakdown field of polyimide films of varying thickness, as reported by Min et al. [127].

The dielectric behavior of polymers is significantly influenced by thickness, particularly as it approaches the nanoscale. Zhang et al. [121] observed that in ferroelectric poly(vinylidene fluoride-trifluoroethylene) (P(VDF-TrFE)) copolymer films, the ε′ sharply declines when the thickness decreases below a critical value of ~100 nm. While polymer dielectrics generally maintain stable ε′ at the microscale, nanoscale films exhibit reduced crystallinity due to spatial confinement. This disruption forces polymer chains into extended conformations, limiting their mobility, thus suppressing crystallization, which collectively degrades dielectric performance. Notably, for films around 1 µm thick, the ε′ remains comparable to that of bulk material, reflecting well-developed crystallinity and stable dielectric properties. This stark transition in ε′ (Figure 10a) below 100 nm indicates that spatial confinement can fundamentally alter the matrix beyond its surface [122].

Building on this, Wang and Yang [123] explored electronic electroactive polymers (EEAPs) and found a strong relationship between thickness and dielectric properties, especially at low frequencies. As thickness decreased, both the ε′ (Figure 10b) and ε″ diminished, attributed to spatial restrictions that limit the rotational freedom and alignment of polymer chains in response to electric fields. This constrained chain mobility reduces dipole flexibility, dampening orientation polarization and leading to lower ε′ and ε″ (Figure 10c). These effects are more pronounced at low frequencies, where interfacial polarization dominates. Together, these findings highlight how thickness-dependent spatial confinement diminishes polarization responses in polymer matrices [124,125,126].

E_BD_ also exhibits a significant thickness dependence, with thinner films often demonstrating enhanced performance due to reduced defect densities. Min et al. [127] studied polyimide films and found the breakdown field (F_b_) decreased with increasing thickness following the inverse power law *F*_b_ = *kd*^−*n*^, where n = 0.324. For instance, F_b_ declined from 639 V/µm at 25 µm to 307 V/µm at 250 µm (Figure 10d). This behavior was linked to space charge accumulation and molecular displacement, which enlarge the free volume under Coulomb forces, enabling electrons to gain energy for breakdown. Simulations based on the charge transport and molecular displacement (CTMD) model supported these results. Similarly, Chen et al. [128] found that in polyethylene, F_b_ followed the same power law (*n* = 0.143) and attributed the decline in E_BD_ with increasing thickness to space charge dynamics enhancing local electric fields to critical strengths of 550 kV/mm. In thinner samples, fewer defects and reduced space charge effects enhanced E_BD_, while surface defects dominated in very thin layers.

Conversely, Thakur et al. [129] demonstrated exceptional E_BD_ in ultra-thin poly(ether methyl ether urea) (PEMEU) films, with values exceeding 1.5 GV/m for films as thin as 1.32 µm. The superior performance of thinner PEMEU films was credited to their high uniformity and reduced defect densities, underscoring the importance of material processing techniques in optimizing dielectric properties. These results collectively emphasize that while thinner films benefit from fewer defects, thus result in enhanced uniformity, the spatial confinement and reduced chain mobility could introduce significant constraints that impact dielectric properties.

### 4.2. Effect of Multilayer Structures

**Figure 11:**
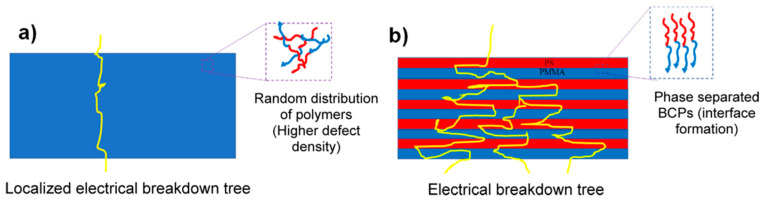
Schematic representation of (**a**) laterally localized breakdown tree in a homogenous film and (**b**) tortuous breakdown pathways in multilayered self-assembled block copolymer films. Adapted with permission from Samant et al. [130], Copyright 2020 American Chemical Society.

Building on the influence of thickness, multilayered polymer films provide additional control over dielectric properties by introducing interfaces that enhance dielectric strength. Studies in the literature have shown that the E_BD_ of homopolymer films improves with increasing molecular weight. This enhancement is often attributed to a lower density of chain ends per unit volume (chain-end density, ρc) or greater structural regularity (chain-end distribution) in polymers with higher molecular weights. The study by Samant et al. [130] investigates the influence of molecular weight and layer thickness on the dielectric E_BD_ of lamellar block copolymer (L-BCP) films, specifically focusing on polystyrene-b-polymethylmethacrylate (PS-b-PMMA). The E_BD_, which is crucial for the energy storage capacity of polymer capacitors, was observed to increase significantly in ordered L-BCP films compared to unordered as-cast films. By using directed self-assembly (DSA) techniques to achieve parallel lamellae, the study shows that these ordered structures yield an E_BD_ up to 50% higher than disordered films. This enhancement is attributed to the layered, interfacial structure in the L-BCP, which hinders the propagation of electrical breakdown pathways, thus offering a “tortuous path” that enhances dielectric strength (Figure 11). The study further examines the effects of molecular weight and chain-end density on dielectric breakdown. Higher molecular weights correlate with increased E_BD_ due to reduced chain-end density, which limits defect sites. The addition of homopolymers to swell the L-BCP domains demonstrated that although thicker layers may increase E_BD_, the introduction of more chain ends with homopolymers could offset this benefit. By exploring these factors, this work provides valuable insights into the design of high-performance dielectric films for flexible electronics and energy storage devices, with recommendations for optimizing molecular weight and layer structure in L-BCPs.

In terms of multilayered films, previous studies have demonstrated that thin alternating layers of polycarbonate (PC) and a poly(vinylidene fluoride-hexafluoropropylene) copolymer (P[VDF-HFP]) exhibit higher dielectric strength (E_BD_) compared to films made from either polymer individually [131]. The ε′ of these multilayer polymers can be reasonably described using an effective medium model; however, the mechanism behind the enhanced dielectric strength remains unclear. A prior article proposed that the increased dielectric strength in these nanolayered materials could be attributed to a “barrier” effect. This phenomenon, well-known in composites, suggests that material boundaries act as barriers, impeding the propagation of dielectric breakdown across the film [132,133,134]. A summary of the effect of polymer structure on dielectric properties is provided in Table 2.

### 4.3. EFM and FIB Techniques to Characterize and Study the Effect of Dielectric Breakdown on Polymer Films

Electrostatic force microscopy (EFM) and focused ion beam (FIB) milling are powerful tools for investigating dielectric properties and breakdown mechanisms in polymer films. These advanced techniques provide detailed insights into nanoscale electrical behaviors and structural changes, enabling a deeper understanding of dielectric performance in various polymer systems.

**Figure 12:**
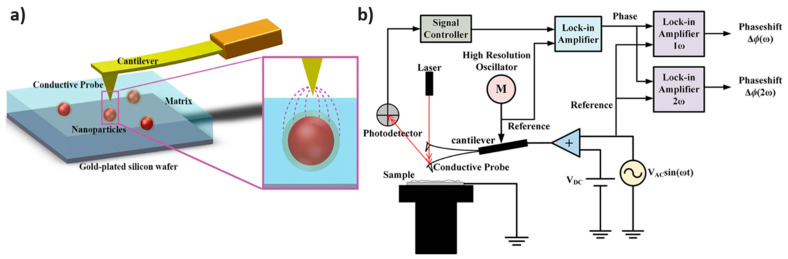
(**a**) Schematic representation of the EFM probe setup used in the experiment. (**b**) Illustration of the principle underlying local dielectric property detection. Adapted from Peng et al. [137].

EFM is a scanning-probe method based on atomic force microscopy (AFM), where the conductive tip is electrically biased, and the effect of electrostatic forces between the tip and sample is detected. EFM can be used to obtain information on various electrical properties of a surface, such as local ε′ at AFM resolutions [135,136]. Peng et al. [137] demonstrated a two-step process for EFM operation to analyze local dielectric properties (Figure 12). First, standard tapping mode imaging maps the surface topography of the sample. This topographical data then guide the probe during a second scan at a fixed lift height above the surface, where an external voltage (V = V_DC_ + V_AC_sin(ωt)) is applied. The resulting electrostatic interactions modulate the probe’s vibration characteristics amplitude, frequency, and phase, providing spatially resolved dielectric measurements. To further enhance accuracy, the study detected the 2ω phase shift signal (Δϕ(2ω)) to mitigate the impact of the work function difference between the probe and the sample. The detection method isolates electrostatic force gradients, which are directly related to the capacitance between the probe and the sample. This approach has been instrumental in advancing the understanding of nanoscale electrical properties in polymer films.

Further extending on this, Gupta et al. [76] investigated the dielectric properties of interfacial regions in polymer nanocomposites using a novel combination of electrostatic force microscopy (EFM) and machine learning (ML). Silica nanoparticles with a diameter of 50 nm, either bare or functionalized with polyaniline (PANI) or PMMA brushes, were embedded in a PMMA matrix to form the nanocomposites. The EFM measurements provided spatially resolved data on the dielectric properties of the nanoparticle interphases. To overcome challenges in directly interpreting EFM data, the researchers trained ML models on finite-element simulations of force gradients. These models allowed for accurate determination of interfacial dielectric constant and thickness. For PANI-functionalized nanoparticles, a distinct interfacial region with higher ε′ was observed, while for PMMA-functionalized particles, the interphase was less pronounced but still detectable.

To investigate the breakdown processes in polymer films, focused ion beam (FIB) milling coupled with scanning electron microscopy (SEM) is applied to produce detailed images of the spatial variations caused by breakdown [138]. FIB milling enables the precise removal of material layers with micrometer and submicrometer (<300 nm) thicknesses. This technique, commonly used for microfabrication in the integrated circuit industry, offers high precision and has proven useful for such analyses. Wolak et al. [131] investigates the dielectric breakdown mechanisms in multilayered polymer films composed of alternating layers of polycarbonate (PC) and a poly(vinylidene fluoride-hexafluoropropylene) copolymer (P[VDF-HFP]). The objective is to understand how layering affects the dielectric strength and breakdown behavior of these materials, which are relevant for high-energy-density applications. Using the FIB-SEM, this study visualizes the changes in the film structure after dielectric breakdown occurs. This approach allows for detailed cross-sectional imaging of the breakdown site, revealing how damage propagates through and between the layers in multilayer films, as compared to homogeneous films.

In homogeneous films, the breakdown damage is confined to a localized pinhole without significant lateral propagation. In contrast, in multilayered films, the breakdown propagates laterally along the interfaces between PC and P[VDF-HFP] layers, extending up to 15 μm from the initial breakdown site. This suggests that the interfaces act as barriers to the breakdown, verifying the enhancement in the dielectric strength through the “barrier effect.”. A summary comparing EFM and FIB is provided in Table 3.

## 5. Dielectric Elastomers and Piezoelectric Polymers

Dielectric elastomers (DEs) and piezoelectric polymers stand out for their unique electromechanical coupling mechanisms, which are highly influenced by chain conformation and nanostructure. This Section explores the properties, mechanisms, and advancements in these materials, emphasizing their impact on performance and functionality.

### 5.1. Dielectric Elastomers

**Figure 13:**
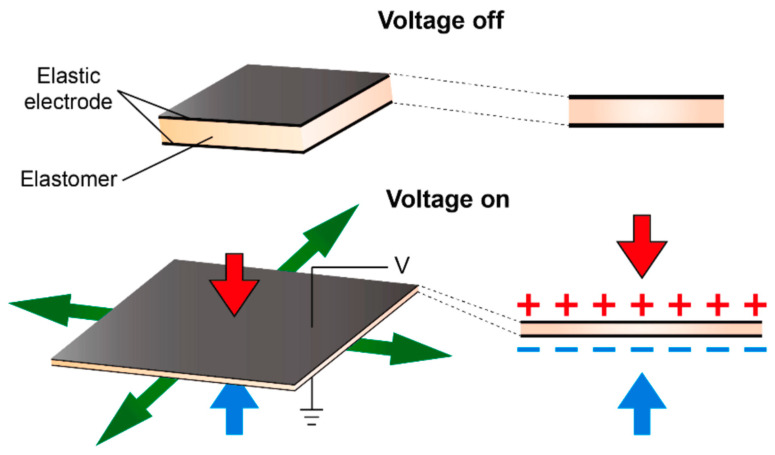
The operating mechanism of the DEA involves two flexible electrodes with an elastomer layer positioned between them. When a high static voltage is applied to the electrodes, the actuator expands in area, while its thickness decreases from the work of Fu al. [164], Copyright 2022 Elsevier, originally reported by Qiu et al. [165].

Dielectric elastomers (DEs) are a class of electroactive polymers capable of significant deformation when subjected to an electric field [144]. They have gained prominence, particularly in soft robotics, due to their ability to produce large actuation strains and high energy densities, comparable to mammalian muscles, across moderate frequencies (up to <1 kHz). DE actuators (DEAs) consist of thin elastomer layers sandwiched between compliant electrodes, leveraging the unique properties of elastomers: softness, high extensibility, and near-incompressibility [145,146]. When a voltage is applied, the elastomer compresses in thickness due to Coulombic attraction while expanding laterally, as illustrated in Figure 13. Their flexibility and stretchability have earned DEAs the nickname “artificial muscles” [147,148], which was initially proposed by Pelrine et al. in 2000 [149,150]. DEAs have since undergone significant development both in materials and design.

Common DE materials include polyacrylates, polyurethanes, slightly polar elastomers (e.g., polyisoprene, hydrogenated nitrile butadiene rubber, ethylene–propylene–diene monomer), and silicone elastomers such as polydimethylsiloxane (PDMS). Despite their advantages, DEs often exhibit low dielectric properties. Researchers have developed various strategies to enhance these properties, including the following:

- Prestretching: Increases breakdown strength and suppresses electromechanical instability [149]. For instance, biaxially prestretched VHB 4910 demonstrated a breakdown strength improvement from 17–31 to 412 V/μm [151,152].
- Blending with polar polymers: Incorporation of materials like polyhexylthiophene (PHT), polyurethane (PU), or polyethylene glycol (PEG) enhances dielectric permittivity. PDMS blends, for example, increased permittivity from 4.6 to 13.8 but showed reductions in breakdown strength and elasticity modulus [153].
- Interpenetrating polymer networks (IPNs): Combining VHB with trimethylolpropanetrimethacrylate (TMPTMA) or silicones with ionic networks improves breakdown resistance and mechanical stability while maintaining prestretch without external forces [154].
- Incorporation of high-ε′ fillers: Additives like ceramics (BaTiO_3_ [154,155,156], TiO_2_ [157,158], or CaCu_3_TiO_12_ [158,159]) or electrically conductive particles (e.g., carbon black [160], graphene [161,162], or conducting polymers [163]) can modify dielectric permittivity and mechanical properties, albeit with trade-offs in breakdown voltage and energy density.

These strategies significantly enhance the performance of DEs, making them adaptable to a range of applications.

#### Liquid Crystalline Elastomer Actuation (LCEs)

Liquid crystal materials, known for their dielectric constant anisotropy, exhibit changes in dielectric constant based on the movement of their molecular polarization under an applied voltage [166]. Among these, liquid crystalline elastomers (LCEs) stand out as a specialized class of shape memory polymers, offering high-stroke, reversible mechanical actuation in response to external stimuli. LCEs are commonly used in fibers or filaments for soft robotics and smart textiles [167,168,169]. For instance, Roach et al. [170] fabricated 3D-printed LCE filaments for thermal management in smart fabrics, while Naciri et al. [171] developed spring-like LCE fibers via melt drawing. He et al. [172] demonstrated LCE microfiber actuators with performance comparable to human muscles.

**Figure 14:**
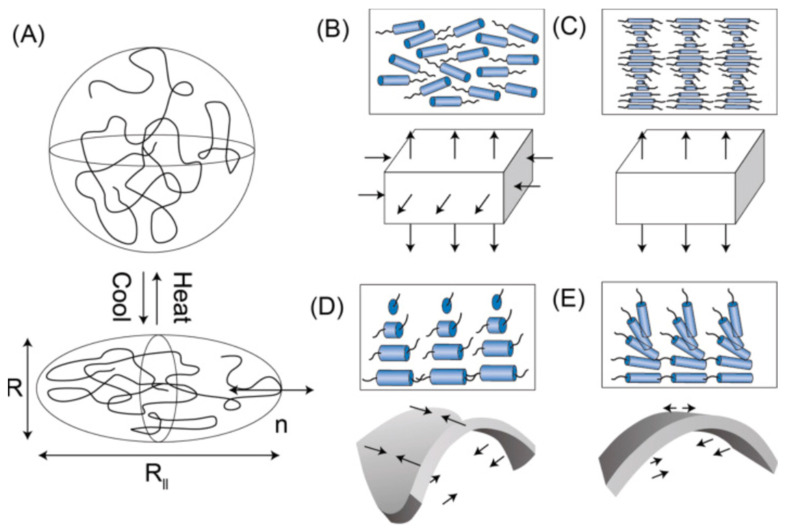
Actuation mechanism of liquid crystalline elastomers (LCEs). (**A**) Thermal stimulation: the nematic phase transitions to the isotropic phase, leading to anisotropic deformation. The shrinkage direction is parallel to the alignment of the LCE fiber or yarn in textiles. (**B**–**E**) Photoinduced deformation: (**B**) anisotropic deformation, (**C**) cholesteric alignment, (**D**) twisted nematic configuration, and (**E**) splay director profiles, with deformations corresponding to a reduction in the order parameter. From Fu et al. [164], Copyright 2022 Elsevier, originally reported by White and Broer [168].

LCEs consist of liquid crystal molecules embedded within polymer chains, with deformation driven by molecular alignment in the elastic network [173]. When exposed to stimuli such as heat or UV light, the LCE transitions between liquid–crystalline and isotropic phases (Figure 14A). Light-driven actuation operates through:

1. Photochemical effect: UV light triggers cis-trans isomerization of azobenzene groups in liquid crystal mesogens, leading to deformation (Figure 14B–E) [174,175].
2. Photothermal effect: A photothermal agent converts light energy into heat, inducing a phase change in the LCE, functioning similarly to heat-driven actuation [176].

These mechanisms make LCEs versatile materials for adaptive systems in textiles and robotics.

### 5.2. Piezoelectric Polymers

Piezoelectric polymers, capable of converting mechanical stress into electrical energy and vice versa, offer advantages such as flexibility, low density, and ease of processing compared to ceramics [177]. They are widely used in sensors, actuators, and energy-harvesting devices. Among them, ferroelectric polymers, particularly those from the polyvinylidene fluoride (PVDF) family, dominate [178,179]. Piezoelectric polymers can either be semicrystalline or amorphous and the mechanisms for each of them differ; however, the piezoelectric effect majorly arises from the alignment of strong molecular dipoles and their response to mechanical stimuli [180,181].

#### 5.2.1. Semicrystalline Piezoelectric Polymers

**Figure 15:**
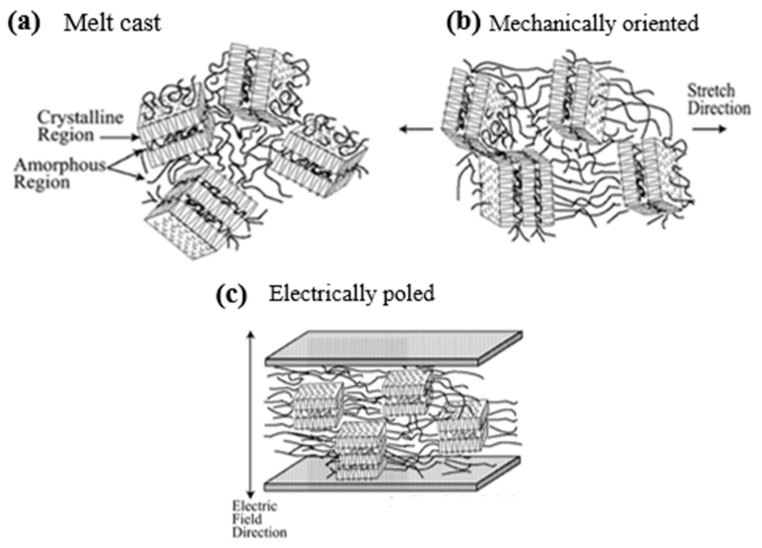
Schematic representation of the structural evolution in PVDF polymer films: (**a**) random stacks of amorphous and crystalline lamellae after melt casting; (**b**) oriented structure after mechanical stretching to several times the film’s original length; (**c**) final morphology after metal electrode deposition and poling through the film thickness, as reported by Harrison et al. [181], Copyright 2002 Wiley.

Semicrystalline polymers gain piezoelectric properties through polar crystalline phases dispersed in amorphous regions (Figure 15a). The amorphous regions determine the polymer’s flexibility, while the crystalline regions define the temperature limits for use. The level of crystallinity depends on how the polymer is processed, with methods like stretching, thermal annealing, or applying a high-voltage field helping to align the crystalline structure. Stretching the polymer aligns the amorphous chains and helps the crystalline regions rotate uniformly when an electric field is applied (Figure 15b). This stretching can be uniaxial or biaxial, which affects whether the material’s properties are uniform or directional. To further align the crystals, a high electric field (around 50 MV/m) is applied, often through direct contact or a corona discharge process, which is commonly used to make PVDF sheets (Figure 15c). The amorphous phase supports the crystalline alignment, and the polarization remains stable over time unless affected by moisture or high temperatures [181]. Examples include PVDF and its copolymers (TrFE, TFE) [182,183], polyamides [184,185,186], polyureas [187,188], and biopolymers [189,190,191].

##### Ferroelectricity in Semicrystalline Polymers

**Figure 16:**
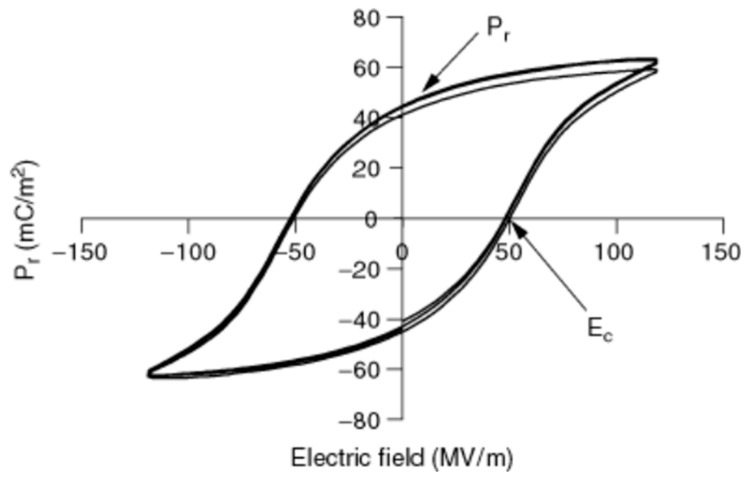
Schematic representation of a typical ferroelectric hysteretic behavior for PVDF as reported by Harrison et al. [181], Copyright 2002 Wiley.

As reported in a study by Harrison and colleagues [181], in semicrystalline polymers like PVDF, polarization shows a nonlinear relationship with the applied electric field under high field conditions, forming a hysteresis loop. This loop, which illustrates spontaneous polarization and its reversibility, is key evidence of ferroelectricity (Figure 16). Two key properties of ferroelectric materials are the coercive field (E_c_), the field strength where the loop crosses the horizontal axis (~50 MV/m at room temperature), and remanent polarization (P_r_), the residual polarization where the loop intersects the vertical axis. Both E_c_ and P_r_ depend on temperature and frequency. The Curie temperature (T_c_), slightly below the polymer’s melting point, marks the transition where the polymer loses its ferroelectric properties above T_c_.

#### 5.2.2. Amorphous Piezoelectric Polymers

Amorphous piezoelectric polymers exhibit a distinct mechanism where polarization arises from the quasi-stable alignment of molecular dipoles during poling [192]. Poling involves applying an electric field at temperatures above the glass transition temperature (T_g_), followed by cooling while maintaining the field. These materials are constrained by operational limits below T_g_ to preserve polarization, although operating near T_g_ can optimize mechanical properties. In contrast, semicrystalline materials maintain stability above T_g_ due to their crystalline structure [193,194,195].

Amorphous piezoelectric polymers are less extensively studied compared to their semicrystalline counterparts, primarily due to their relatively low piezoelectric responses, which have yet to meet the threshold for significant commercial interest. Much of the research in this area has focused on nitrile-substituted polymers, including materials such as polyacrylonitrile (PAN) [196,197,198], polyphenylethernitrile (PPEN) [199,200], poly(vinylidene cyanide vinyl acetate) (PVDCN/VAc) [201,202,203,204], and poly(1-bicyclobutanecarbonitrile) [205]. Additionally, weak piezoelectric effects have been observed in polymers like polyvinyl chloride (PVC) and polyvinyl acetate (PVAc) [194,206,207,208]. Among these, vinylidene cyanide copolymers show the greatest potential, demonstrating high dielectric relaxation strengths and relatively strong piezoelectric properties.

## 6. Conclusions

This review underscores the profound impact of polymer chain conformation and nanostructure on dielectric properties, consolidating knowledge across critical aspects such as free volume, crystallinity, and chain rigidity. The interplay between molecular conformation and dielectric performance, particularly in high-energy applications emphasizes the need for meticulous molecular design and optimization. Advances in polymer blending, nanofillers, and multilayer structures have demonstrated significant potential in enhancing ε′, reducing ε″, and improving E_BD_. However, several limitations and knowledge gaps remain.

Key Challenges and Limitations:

- Complex Interplay Between Conformation and Properties: The relationship between polymer chain conformation and dielectric properties is highly complex, involving multiple interdependent factors such as free volume, chain rigidity, crystallinity, and dipole mobility. Systematic understanding and isolating the contributions of each factor remains a daunting task due to the synthetic challenges and lack of a model system.
- Lack of Unified Theories: Existing studies often present conflicting findings, particularly regarding the optimal balance between chain flexibility and rigidity for achieving high ε′ while minimizing ε″. The absence of a unified framework or model to predict these behaviors under varying conditions limits progress in this area.
- Material Design Trade-offs: Enhancing one dielectric property (e.g., ε′) often compromises others (e.g., E_BD_ or ε″). Designing polymers with a balanced combination of high ε′, low ε″, and high E_BD_ is a persistent limitation.
- Processing and Scalability Issues: Polymer-processing techniques, such as spin coating or extrusion, can significantly influence chain conformation. However, achieving consistent and scalable processing methods that preserve desired conformations across different scales is a major challenge, particularly for nanocomposites.
- Environmental and Operational Stability: Polymers in real-world applications must withstand harsh conditions, including high temperatures, moisture, and mechanical stress. The influence of environmental factors on chain conformation and dielectric performance is not fully understood, limiting long-term reliability.
- Limited Exploration of Conformation–Nanocomposite Interactions: while studies have highlighted the role of chain conformation in nanocomposites, the exact mechanisms by which polymers interact with fillers at the interfacial level and how these interactions impact dielectric properties need deeper investigation.

Research Priorities and Actionable Directions:

To address these challenges and unlock the full potential of polymer dielectrics, future research should prioritize the following directions:

- Development of Predictive Theoretical Models: Establish a unified framework to quantify the relationship between chain conformation, nanostructure, and dielectric properties. Integrating computational modeling and machine learning approaches can accelerate predictions and material optimization.
- Systematic Molecular Engineering: Focus on designing polymers with tunable chain conformations to achieve balanced dielectric properties. For example, tailoring interchain interactions through molecular design or using side groups to control dipole alignment.
- Innovative Processing Techniques: Explore advanced processing methods such as directed self-assembly, electric field alignment, or additive manufacturing to preserve desired chain conformations and enhance scalability. Developing in situ characterization tools to monitor chain conformation during processing will further optimize results.
- Enhanced Stability Solutions: Investigate the use of additives, crosslinking agents, or surface modifications that stabilize polymer conformation against environmental degradation. This includes developing polymers with intrinsic resistance to moisture, temperature, and mechanical stress.
- Deeper Analysis of Interfacial Interactions: Conduct focused studies on polymer-filler interfacial dynamics to elucidate how nanofillers influence chain conformation and dielectric performance. Advanced characterization techniques, such as in situ atomic force microscopy (AFM) and transmission electron microscopy (TEM), should be employed to visualize these interactions at the nanoscale.
- Real-World Application Testing: Develop testing protocols to evaluate polymer dielectrics under operational conditions such as those found in electric vehicles, flexible electronics, and high-capacity capacitors. This approach will bridge the gap between laboratory research and industrial applications.
- Sustainability and Cost-Effectiveness: Explore environmentally friendly polymers and scalable production methods to meet the increasing demand for sustainable materials in energy storage and electronics.

### Final Outlook

By addressing these priorities, the scientific community can make significant strides in advancing polymer dielectrics for next-generation technologies. These efforts will enable the development of high-performance, durable, and cost-effective materials tailored to the growing demands of energy storage, flexible electronics, and beyond. Unlocking the potential of polymer dielectrics promises to drive transformative advancements across industries, from renewable energy systems to wearable electronics.

## Figures and Tables

**Table 1:** Generalized view of dielectric properties of polymers and their nanocomposites.

Property	Polymers	Nanocomposite	References
Dielectric Constant (ε′)	Typically low in most polymers (usually ε′ < 4.5, except PVDF). Enhanced with dipolar groups in polar polymers.	Significantly increased with nanofillers. Particularly high-ε′ fillers like BaTiO_3_, BST, or Al_2_O_3_.	[11,16,19,65,66,67,69,70,71,72,73,74,75,76,80,81,82,83,84,96,97,98]
Dielectric Breakdown Strength (EBD)	Generally stable in pure polymers.	Decreases at high nanofiller concentrations (>20%) due to inhomogeneous electric fields and localized defects.	[76,103,104]
Dielectric Loss (ε″)	Typically low in pristine polymers.	Increased with high filler content or poor dispersion but optimized with low concentration nanofillers.	[110,113,114]
Energy Storage Capability	Moderate energy density in pristine polymers due to low ε′ and EBD.	Enhanced energy density through nanofillers, with better performance at optimized filler sizes, shapes, and low concentrations (<10%).	[96,97,98,104,105]

**Table 2:** Summary of structural modification of polymers, improvement, and deterioration of dielectric properties.

S/N	Structural Modification	Improvement in Dielectric	Deterioration in Dielectric Properties	References
1	Increase in free volume via rigid backbone (e.g., SO_2_-PPO)	Increased ε′ due to improved dipole alignment (ε′ = 8.2)	Increased dipole rotation and mobility leads to decrease in ε″.	[40,44]
2	Blending PEI and PVH	Higher ε′ from dipole alignment due to PVH groups	Limited ε′ at high PEI content due to constrained dipole mobility.	[45]
3	Methyl side groups	None observed	Increased free volume lowers ε′	[22]
4	Rigid polymer chains (e.g., PPO)	Reduced ε″ due to restricted dipole mobility	Minimal ε′, reduced flexibility.	[52]
5	Nanofillers with high ε′ (e.g., BaTiO_3_, CNFs, BST)	Higher ε′, enhanced interfacial polarization	Reduced E_BD_ due to inhomogeneous fields at high filler volume.	[75,108]
6	Use of low-ε′ fillers (e.g., alumina)	Improved ε′ with stable E_BD_	None observed.	[104]
7	Smaller nanoparticle sizes (<20 nm)	Enhanced ε′ due to larger interfacial regions	None observed.	[104]
8	1-D nanorods in nanocomposites	Significant ε′ improvement due to extended interfacial regions	None observed.	[105]
9	Blending PI with PEI	Higher E_BD_, reduced void density (E_BD_ ~1000 MV/m)	None observed.	[28]
10	Cyclic polystyrene (cPS) vs. linear polystyrene	50% enhancement in EBD, 80% increase in energy density	None observed.	[55]
11	Multilayered structures (e.g., PC and P[VDF-HFP])	Increased E_BD_ via barrier effect	None observed.	[130,131]
12	Nanoconfinement	Increased E_BD_ and ε″	Reduction in ε′.	[121,122,123,127]
13	Polymer chain annealing	Improved E_BD_ and reduced ε″	None observed.	[64]

**Table 3:** Summary of EFM and FIB.

Technique	Description	Advantages	Limitations	References
Electrostatic Force Microscopy (EFM)	Measures electrostatic interactions between the tip and the sample, often used for dielectric materials.	Provides information on charge distribution and dielectric properties.	Requires precise calibration, sensitive to environmental noise.	[135,136,137,139,140,141]
Focused Ion Beam (FIB)	Uses a beam of ions to modify or analyze materials at the nanoscale, often combined with SEM.	Enables precise milling and sectioning for subsurface analysis.	Potential for ion damage to samples, complex and expensive equipment.	[131,138,139,142,143]
